# The Most Frequent Psychiatric Comorbidities in Bulgarian Patients With Epilepsy: Their Connection With the Main Clinical Characteristics and Quality of Life

**DOI:** 10.7759/cureus.66149

**Published:** 2024-08-04

**Authors:** Irina Vaneva, Rumyana Kuzmanova, Katerina Stambolieva

**Affiliations:** 1 Department of Epilepsy, Multiprofile Hospital for Active Treatment in Neurology and Psychiatry “St. Naum”, Sofia, BGR; 2 Department of Neurology, Medical University, Akad, Sofia, BGR; 3 Department of Cognitive Psychophysiology, Bulgarian Academy of Science, Institute of Neurobiology, Sofia, BGR

**Keywords:** quality of life, adverse drug effects, seizure frequency, psychiatric comorbidity, epilepsy

## Abstract

Aim: The objective of this study is to determine and compare the relationship of the most common psychiatric comorbidities in Bulgarian patients with epilepsy with the main clinical characteristics, as well as to evaluate their impact on certain aspects of the quality of life.

Clinical rationale: Psychiatric comorbidities occur in about one-third of people with epilepsy throughout their lifetime, and their incidence is much greater in high-risk groups such as patients with treatment-resistant epilepsy.

Material and methods: The study group consisted of 129 participants, of whom 104 were divided into four groups according to the presence of one of the most frequently diagnosed psychiatric comorbidities in our patients with epilepsy: personality and behavioral disorder (PBD) (n=25), mild to moderate depressive disorder (n=26), anxiety disorder (n=32), and dissociative and conversion disorders (n=21). A control group was also formed with a similar number of participants with epilepsy (n=25) without psychiatric comorbidity. Some sociodemographic and clinical characteristics of epilepsy were analyzed in all patients. All patients filled out two questionnaires: the Bulgarian version of quality of life in epilepsy - 89 (QOLIE-89) and the Bulgarian version of SIDAED (assessing SIDe effects in antiepileptic drugs (AED) treatment).

Results: The analysis revealed a negative influence of psychiatric comorbidity on the presence of epileptic seizures, unwanted drug effects, and lower scores for all aspects of the quality of life of patients with epilepsy.

Conclusion and clinical implications: The main conclusion of our study is the presence of an interaction between psychiatric comorbidity, the clinical course of the disease, and the deteriorated quality of life (QOL) in patients with epilepsy. Further attention, comprehensive care, and targeted research are needed to analyze individual psychiatric comorbidities in patients with epilepsy for early detection and treatment.

## Introduction

Psychiatric comorbidities occur in about one-third of people with epilepsy throughout their lifetime [1]. Theories about the comorbidity of epilepsy and psychiatric disorders are complex, and recurrent associations between these conditions suggest an overlap that is more than just always co-occurring [2]. Mental disorders affect the quality of life (QOL) of patients with epilepsy to a greater extent than the seizures themselves [3,4].

Depressive disorders (DD) are the most common psychiatric comorbidity in patients with epilepsy, with a lifetime prevalence 30-35% higher than in the general population [3], and a complex relationship exists between epilepsy and DD. DD and anxiety disorders (AD) often co-occur [5] and their comorbidity is associated with the occurrence of more severe adverse effects (AEs) and worse tolerability of antiseizure medications (ASMs) [5]. A cohort study using data from the UK General Practice Research Database showed that the incidence of AD was not only higher in people with epilepsy compared to controls but was already increased 3 years before the diagnosis of epilepsy [4]. A personality and behavioral disorder (PBD) may be a residual or concomitant disorder of brain damage or dysfunction and is characterized by a significant alteration of the usual premorbid behavior, including the expression of emotions, needs, and motivation. Dissociative and conversion disorders (DCD) are relatively common in patients with epilepsy, and they often have dissociative seizures in addition to preexisting epileptic seizures (ES). About 5-10% of all patients with confirmed dissociative seizures are also diagnosed with ES [6]. In people with epilepsy, psychiatric comorbidities have been historically associated with poor QOL [1]

The purpose of this study is to determine and compare the relationship of the most common psychiatric comorbidities in Bulgarian patients with epilepsy with the main clinical characteristics as well as to evaluate their impact on certain aspects of the QOL.

## Materials and methods

The study group consisted of 129 patients with epilepsy who were divided into four groups according to the presence of any of the most frequently diagnosed psychiatric comorbidities: PBD (n=25), mild to moderate DD (n=26), AD (n=32), and DCD (n=21), and a control group of participants with epilepsy (n=25) who lacked psychiatric comorbidity. The screened participants are the patients with epilepsy in the Epilepsy Department of the University Hospital of Neurology and Psychiatry “St. Naum,” Sofia, Bulgaria. The psychiatric assessment was administered by a psychiatrist with extensive experience in epilepsy and psychiatric disorders according to the International Classification of Diseases 10th Revision (ICD-10) [7]. Patient selection was based on the following additional criteria: (1) epilepsy diagnosed according to the International League Against Epilepsy (ILAE) criteria for more than 1 year; (2) age over 18 years; (3) stable doses of ASMs for at least the last 3 months before the study entry; and (4) patients with other chronic progressive neurologic, severe psychiatric, or somatic disease were excluded from the study.

In all patients, basic sociodemographic factors such as gender, age, family status, education, and professional employment were analyzed, as well as certain clinical characteristics of the epileptic disease - type of epilepsy according to the etiology of the disease (genetic generalized, focal, with unclear etiology), duration of epilepsy, average monthly frequency of ES (no seizures, 1-2, 3, and more), number of ASMs (1, 2, 3, and more medications), AEs when taking ASMs, presence of focal neurological symptoms (focal symptoms), compliance of the patient.

All patients filled out two questionnaires: the Bulgarian version of the QOL in epilepsy - 89 (QOLIE-89) [8] and the Bulgarian version of the SIDAED (assessing SIDe effects in antiepileptic drugs (AED) treatment) questionnaire [9].

QOLIE-89 is a qualitative method for determining QOL in the general population of patients with epilepsy and consists of 89 questions divided into 17 domains: seizure worry, medication effects, health perceptions, health discouragement, work/driving/social function, language, attention/concentration, memory, overall QOL, emotional well-being, role limitations: emotional, role limitations: physical, social isolation, social support, energy/fatigue, physical functioning, and pain. The values for each subscale were calculated by using specially designed tables and formulas [8]. Higher values indicate better QOL.

The SIDAED-BG scale is a specific questionnaire for the study of AEs in ASMs, recommended to assess the severity of side effects possibly related to anticonvulsant drugs. It consists of 46 questions related to adverse drug reactions, distributed in nine domains: general CNS, behavior (increased irritability), depressive symptoms, cognitive function, motor problems and coordination, visual complaints, headache, cosmetic and dermatological complaints, gastrointestinal complaints, and sexuality and menses [10]. Higher values indicate more adverse drug reactions in patients with epilepsy.

All patients were volunteers and signed informed consent to participate in the study approved by the ethics committee of multiprofile hospital for active treatment in neurology and psychiatry “St. Naum” (IRB №7/16.Nov.2022) and in accordance with the ethical standards of the Declaration of Helsinki.

Statistical methods

Descriptive statistics was used for the calculation of demographic, clinical data, and scores of psychometric scales SIDAED-BG and QOLIE-89. The Kolmogorov-Smirnov test was used to evaluate the normality of distribution and determination of appropriate statistical methods. Continuous variables were presented as the mean value ± standard deviation (SD) and were analyzed with independent sample T-test, one-way ANOVA, and the post-hoc Newman-Keuls tests. Categorical variables were described by frequencies (percent) and chi-square tests were used to identify associations between groups. Spearman’s correlation was applied to evaluate the correlation between SIDAED scores and patients’ clinical characteristics. The strength of the correlation was determined as follows: correlation coefficients between 0.1 and 0.2 indicate a weak correlation, coefficients between 0.3 and 0.5 indicate a fair correlation, coefficients between 0.6 and 0.7 indicate a moderate correlation, and coefficients between 0.8 and 1 indicate a strong correlation [11,12]. The level p ≤ 0.05 was accepted as statistically significant (Statistica 8.0 for Windows, StatSoft Inc., USA).

## Results

Table 1 presents the sociodemographic and clinical characteristics of the four groups of patients with epilepsy and psychiatric comorbidity and the control group of participants (Group 5), the overall assessment from the SIDAED-BG and QOLIE-89 questionnaires completed by them, as well as the significant statistical differences established between the groups.

**Table 1:** Sociodemographic, clinical characteristics, and general assessment from SIDAED-BG and QOLIE-89 questionnaires Data are presented as means ± SD or number of subjects and percentage. **p<0.001, *p<0.05: statistical significance differences between groups of patients with psychiatric comorbidity (G1-G4) and control group of patients without psychiatric comorbidities (G5) (Newman-Keuls tests). Groups: 1- personality and behavioral disorder (PBD); 2- mild to moderate depressive disorder (DD); 3- anxiety disorder (AD); 4- dissociative and conversion disorders (DCD); and 5- control group of participants with epilepsy without psychiatric comorbidity. Domains of the SIDAED-BG questionnaire: D1- general CNS; D2- behavior/increased irritability; D3- depressive symptoms; D4- cognitive function; D5- motor problems and coordination; D6- visual complaints; D7- headache; D8- cosmetic and dermatological complaints; and D9- gastrointestinal complaints. Domains of the QOLIE89-BG questionnaire: D1- seizure worry; D2- medication effects; D3- health perceptions; D4- health discouragement; D5- work/driving/social function; D6- language; D7- attention/concentration; D8- memory; D9- overall QOL; D10- emotional well-being; D11- role limitations: emotional; D12- role limitations: physical; D13- social isolation; D14- social support; D15- energy/fatigue; D16- physical functioning; and D17- pain.

Parameters		Group 1 (n=25)	Group 2 (n=26)	Group 3 (n=32)	Group 4 (n=21)	Group 5 (n=25)
Age (years) Mean±SD		39.52±12.73	42.65±13.72	37.61±12.73	36.57±13.15	44.12 ±14.33
Duration of disease (years) Mean±SD		14.84±8.01	17.46 ±9.33	17.72±8.79	14.47±10.55	13.08±9.53
Gender N (%)	Males	13(52)	9(35)	15(47)	9(43)	9(36)
	Females	12(48)	17(65)	17(53)	12(57)	16(64)
Marital status N (%)	Married	9(36)	21(81)	20(63)	13(62)	17(68)
	Alone	16(64)	5(19)	12(37)	8(38)	8(32)
Employment N (%)	Working	13(52)	17(65)	24(75)	13(62)	15(60)
	Non-working	12(48)	9(35)	8(25)	8(38)	10(40)
Education N (%)	Primary	0 (0)	2(8)	4(13)	2(10)	4(16)
	Secondary	18(72)	12(46)	11(34)	14(66)	13(52)
	Higher Secondary	7(28)	12(46)	17(53)	8(24)	8(32)
Epilepsy N (%)	Genetic generalized epilepsy	0(0)	6(23)	9(28)	5(24)	5(20)
	Focal epilepsy	3(12)	15(58)	15(47)	11(52)	15(60)
	Unclear	22(88)	5(19)	8(25)	5(24)	5(20)
Seizure frequency N (%)	Seizure free	5(20)	9(35)	12(37)	12(57)	14(56)
	2 per month	17(68)	13(50)	15(47)	6(29)	10(40)
	≥3	3(12)	4(15)	5(16)	3(14)	1(4)
Focal symptoms N (%)	Without	2(8)	13(50)	19(59)	9(43)	19(76)
	With	23(92)	13(50)	13(41)	12(57)	6(24)
AEMs intake N (%)	1	3(12)	9(35)	10(31)	11(52)	13(52)
	2	9(36)	10(38)	17(53)	7(34)	9(36)
	≥3	13(52)	7(27)	5(16)	3(14)	3(12)
Compliance N (%)	Yes	13(52)	19(73)	21(66)	15(71)	21(84)
	No	12(48)	7(27)	11(34)	6(29)	4(16)
SIDAED-BG total scores		56.4±16.4**	56.1±13.2**	57.8±11.8**	53.5±16.5**	35.2±12.5
QOLIE total scores		55.6±9.9*	58.1 ±10.7*	57.02±10.7*	57.1 ± 12.7*	67.6 ± 8.9

Sociodemographic characteristics

The average age of the studied patients was 40.19±13.41. No statistically significant difference was found in the age of patients from the five formed groups (Table 1). As shown in Table 1, women predominate in all groups, except the group of patients with PBD (Group 1). Regarding professional employment and education, working participants and those with secondary education predominate. No statistically significant difference was found in the percentage ratio for the indicators of gender and professional employment between the five groups of patients (χ2 test, p<0.05). Statistically significant differences were found in the ratio of the family to non-family patients in Groups 1 and 2, and in Group 1 there were significantly fewer family patients compared to the other groups, and in Group 2 the ratio was the opposite (Table 1).

Clinical characteristics

The duration of epilepsy of the studied patients was 15.68±9.25, and no significant differences were found between the individual groups (Newman-Keuls tests, p<0.05) (Table 1). In all groups of subjects, the participants with good compliance to the treatment predominated (52-84%), with the highest percentage in the group without psychiatric comorbidity and the lowest in patients with PBD. Regarding the type of epilepsy indicator, only Group 1 differed significantly from the others, with patients with epilepsy of unknown etiology predominating. In the patients with PBD, participants with focal neurological symptoms significantly predominated (92%), while in the control group, most of the patients had a normal neurological status (76%). More than 50% of patients without psychiatric symptoms and patients with DCD were found to be without ES and receiving 1 ASM, in contrast to the other three groups. In patients without psychiatric comorbidity, only 4% had a monthly seizure frequency of ≥3, in contrast to the other groups with psychiatric comorbidity, in which over 10% had more than three seizures per month. The proportion of patients receiving ≥3 ASMs was highest in the PBD group (52%), and lowest in the control group (12%).

AEs in ASMs Intake

AEs in ASMs intake were assessed with the SIDAED-BG questionnaire. From the presented data on the total score of the questionnaire on the severity of AEs, a statistically significantly lower total score was found in the control group of patients without psychiatric comorbidity compared to the others, while significant differences between the other groups of patients with psychiatric comorbidities were not identified (Newman-Keuls tests, p<0.05) (Table 1).

**Figure 1:**
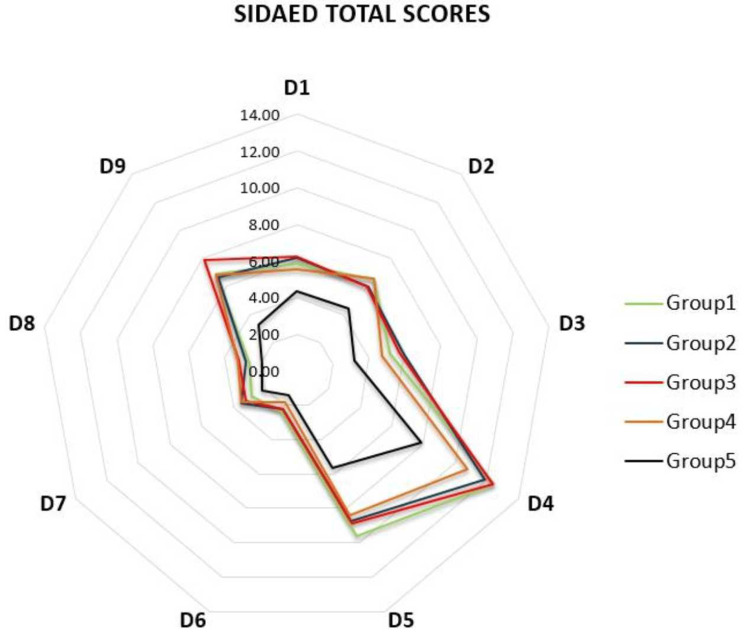
Mean values of the total scores of the nine domains of the SIDAED-BG questionnaire for all investigated patients’ groups. Groups: 1- personality and behavioral disorder (PBD); 2- mild to moderate depressive disorder (DD); 3- anxiety disorder (AD); 4- dissociative and conversion disorders (DCD); and 5- control group of participants with epilepsy without psychiatric comorbidity. Domains: D1- general CNS; D2- behavior/increased irritability; D3- depressive symptoms; D4- cognitive function; D5- motor problems and coordination; D6- visual complaints; D7- headache; D8- cosmetic and dermatological complaints; and D9- gastrointestinal complaints.

Figure 1 presents a radar diagram reflecting the comparison of the average values in the nine subdomains of the SIDAED-BG questionnaire for the five studied groups of patients, with higher values indicating greater severity of adverse drug effects.

The group without psychiatric comorbidity (Group 5) had a statistically significant lower mean score compared to the other four groups of patients, while no differences in the mean domain score between groups with psychiatric comorbidity were found. The results are as follows: D1 (general CNS) mean score of Group 5 was 4.32±1.89, and for the others between 5.57±2.25 and 6.25±1.58; D2 (behavior) average score for Group 5 is 4.40±2.60, for groups from 1 to 4 between 6.00±2.01 and 6.57±2.62; D3 (depressive symptoms) mean score for Group 5 was 3.20±1.85, and for the rest between 4.76±1.67 and 5.88±1.82; D4 (cognitive function) mean score for Group 5 was 4.40±2.60, and for the others it was 2.5 to 3 times higher 10.81±5.15 and 12.44±4.13; D5 (motor problems and coordination) for the control group was 5.64±3.15, and for the psychiatric comorbidity groups the mean value was between 8.43±3.40 and 9.60±3.74. For the gastrointestinal complaints domain, the mean score of the groups with psychiatric symptoms was between 6.86±2.57 and 7.86±2.32, significantly higher than those of the control group (3.28±1.72). For the other domains, no statistically significant differences were found.

A significant positive correlation was established between the frequency of ES and the mean score of D5 for Group 1 (rs=0.4), Group 2 (rs=0.46), and Group 4 (rs=0.45), and mean score of D2 and D4 for Group 4 were rs=0.41 and rs=0.42, respectively. Only in Group 4 a significant positive moderate correlation between the frequency of seizures and the mean score of D1 (rs=0.62) was observed.

Analyzing the average values of the total score of the SIDAED-BG questionnaire depending on the frequency of ES in the five groups of patients (Mann-Whitney U test, p<0.05) a statistically significant difference was found between the group without psychiatric symptoms and all patients with psychiatric comorbidity, regardless of ES frequency. With more than three seizures per month, a statistically significant difference was found between patients with AD and those with DCD, who also showed the highest total value of the SIDAED-BG scale (70.3±19.4).

Quality of Life

From the presented data on the total value of the QOLIE-89 questionnaire, a significantly higher total score was found in the control group of patients with epilepsy without psychiatric comorbidity compared to the others.

**Figure 2:**
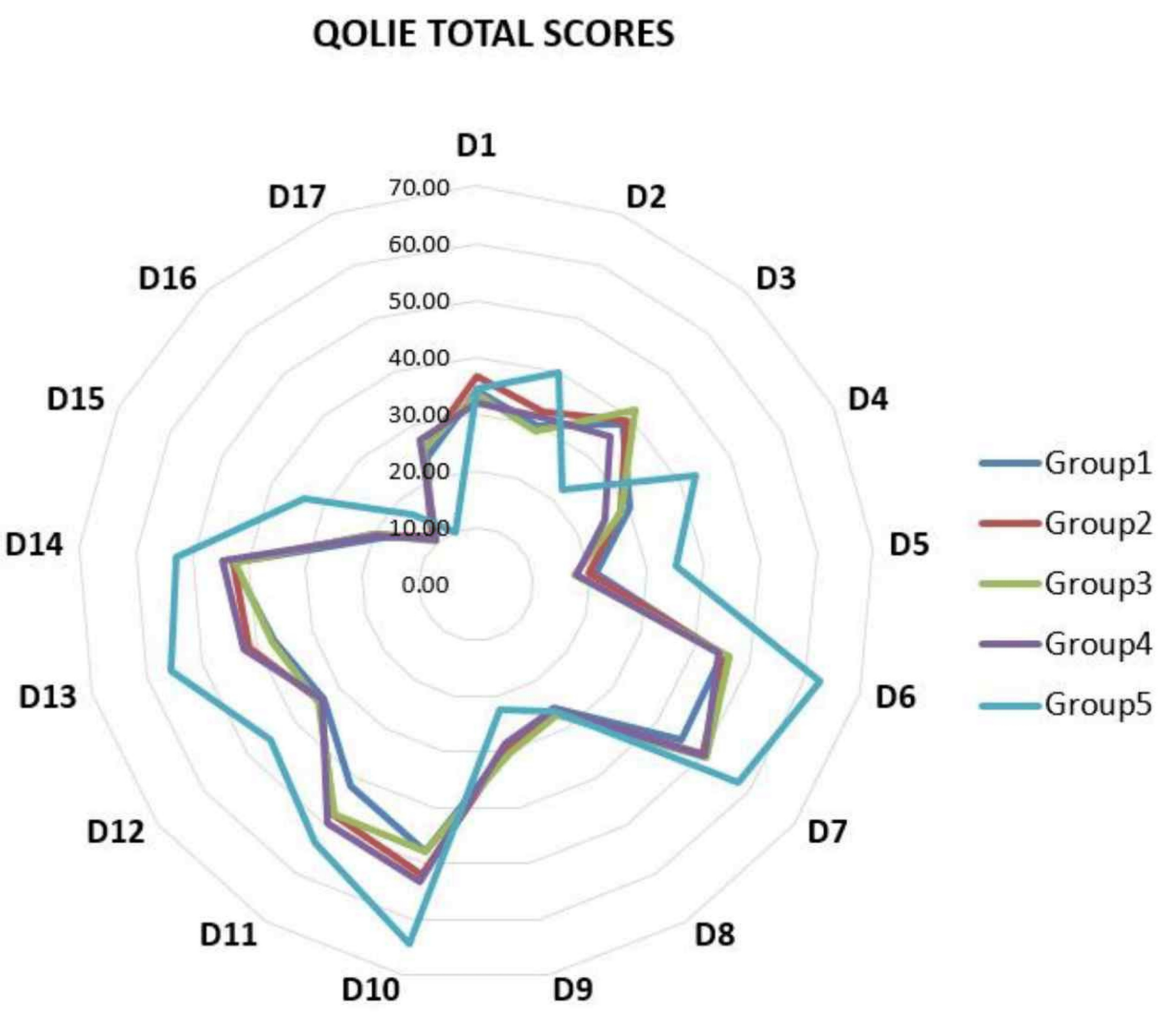
Mean values of the total scores of the 17 domains of the QOLIE89-BG questionnaire for all investigated patients’ groups. Groups: 1- personality and behavioral disorder (PBD); 2- mild to moderate depressive disorder (DD); 3- anxiety disorder (AD); 4- dissociative and conversion disorders (DCD); and 5- control group of participants with epilepsy without psychiatric comorbidity. Domains: D1- seizure worry; D2- medication effects; D3- health perceptions; D4- health discouragement; D5- work/driving/social function; D6- language; D7- attention /concentration; D8- memory; D9- overall QOL; D10-emotional well-being; D11- role limitations: emotional; D12- role limitations: physical; D13- social isolation; D14- social support; D15- energy/fatigue; D16- physical functioning; and D17- pain.

The presented radar chart (Figure 2), reflects the comparison of the mean values in the 17 subdomains of the QOLIE-89 questionnaire, it is clearly seen that the QOL scores of patients with epilepsy in the group without psychiatric comorbidity are the highest except for the domains D17 (social isolation), D3 (physical function), and D9 (emotional well-being).

When comparing ES frequency and QOLIE-89 questionnaire values in the group of patients without psychiatric comorbidity, there was a statistically significant decrease in the total score from 70.2±9.3 for patients without ES to 40.1±11.2 for those with 2-3 ES monthly, showing a dramatic deterioration in QOL with increasing seizure frequency. In patients with psychiatric comorbidities, a more marked deterioration in QOL was observed for the groups with DD and those with DCD at a monthly frequency of ES greater than 3, compared to patients with the same psychiatric comorbidities but without ES.

For Group 4, a significant fair negative correlation was observed between the number of ES and the average values of the subscales of the QOLIE-89 questionnaire: D6 (rs=-0.40), D7 (rs=-0.57), D8 (rs=-0.43), D10 (rs=-0.54), D12 (rs=-0.51), D13 (rs-0.44), and D15 (rs=-0.43).

## Discussion

This is the first systematic research in Bulgaria to assess the course of epileptic disease depending on the presence of psychiatric comorbidities in the general population of patients with epilepsy. The objective of our study was to determine and compare the interrelationship of the most common psychiatric comorbidities in Bulgarian patients with epilepsy and the main clinical characteristics of the disease. We assessed their impact on the perception of the severity of AEs when using ASMs and certain aspects of QOL with the help of validated and adapted Bulgarian-language-specific questionnaires.

Many authors have discussed the negative impact of psychiatric comorbidity on the clinical characteristics and QOL of patients with epilepsy. Epilepsy is a heterogeneous disease and thinking of comorbidity between two etiologically unrelated conditions would be inaccurate [2]. As shown by the presented results the presence of psychiatric comorbidity, as an independent predictor, has a statistically significant negative effect on the course of the disease (occurrence of AEs, frequency of ES, number of taken ASMs) and QOL in patients with epilepsy. Unfortunately, when analyzing the results, we did not find statistically significant differences between patients with different forms of psychiatric comorbidity for many of the clinical characteristics including duration and etiology of epilepsy. The greatest difference was found between patients with PBD and the control group regarding the presence of focal symptoms, patient compliance with treatment, and etiology of epilepsy.

The largest number of our patients without ES was in the group without psychiatric comorbidity (n=14). These patients respectively are treated with 1 ASM (n=13), especially compared to patients with epilepsy and PBD. The ratio was reversed for patients with ≥3 ES monthly and receiving ≥3 ASMs. According to other authors, psychiatric comorbidities are associated with a fourfold increased risk of drug resistance in focal [13] and generalized epilepsy [14]. This was confirmed by a large cohort study in Italy of 1006 people with newly diagnosed epilepsy observed for an average of 16 years, showing that the absence of psychiatric comorbidities predicted early and long-term freedom from ES [15]. Although psychiatric comorbidities have been found to be more common among patients with pharmacoresistant epilepsy, the literature also lacks data on whether the type and severity of psychiatric comorbidities differ in their association with seizure control [13].

From the presented results when comparing the mean values in the nine subdomains of the SIDAED-BG questionnaire, it is clear that the total AEs severity score in the group without psychiatric comorbidity is the lowest. In our study, the most severe AEs were observed for the domain “Cognitive Function.” The impairment of cognitive functions in the presence of psychiatric comorbidity has also been established by other authors [16,17]. Salas-Puig et al. found a significant correlation between psychiatric symptoms and cognitive impairment in 661 patients with epilepsy [16]. According to the authors, these facts, on one hand, necessitate the assessment of the level of anxiety and depression in all patients with epilepsy, especially in thоse with serious cognitive complaints, and on the other, an objective psychiatric and neuropsychological examination of these patients. According to Stephen et al., psychiatric comorbidities are associated with a high risk of side effects, especially cognitive complaints and psychiatric side effects [17]. In clinical practice, the presence of more AEs in patients with psychiatric comorbidity could lead to an unnecessary change in the regimen of antiepileptic treatment. The perception of AEs from antiepileptic treatment increased significantly with a greater frequency of ES [9]. We found a statistically significant difference for AEs depending on the average monthly frequency of ES in the five groups of patients, as well as between the group without psychiatric symptoms and all patients with psychiatric comorbidity, regardless of the frequency of ES. In patients with well-controlled ES, statistically significantly the most pronounced AEs were patients with depression and AD. Patients with more than three seizures per month with the highest total value on the SIDAED-BG scale were the groups with anxiety and those with dissociative disorder. Similar results were found in a study by Asadi-Pooya et al., where anxiety exists as a frequent comorbidity in patients with epilepsy, and increases with frequency of ES [18]. Although similar results have been reported in studies with different methodologies, depression is clearly higher in pharmacoresistant epilepsy [19,20].

Our finding of a negative effect of psychiatric comorbidity in patients with epilepsy on QOL is not surprising. It is well known that QOL in people with epilepsy is significantly impaired [21,22]. In our patients with epilepsy, the available psychiatric symptomatology significantly affected the evaluation of the various aspects of QOL, as the dependence between QOL and the presence of psychiatric comorbidity was inversely proportional, and the limitations related to the subscales “social support,” “social isolation,” “role limitations: emotional,” “energy/fatigue” were most significantly affected. The mean scores of the QOLIE-89 domains were significantly lower in patients with psychiatric comorbidity compared to those without, except for emotional well-being, social isolation, and physical function. Epilepsy-related factors have been proven to have a more limited impact on the QOL of epilepsy patients compared to mood disorders (anxiety and depression), which have the most significant impact in these patients [23] while other authors do not fully support these claims [24]. A high incidence of ES limits usual daily activities and leads to deterioration of physical, social, and emotional functioning, and ultimately to deterioration of overall QOL. Seizure frequency is the most important determinant of QOL, both in drug-resistant epilepsy and in groups of patients with well-controlled ES [25,26]. In all groups of our patients, deterioration of QOL with increasing frequency of ES was observed, most pronounced for the group without psychiatric comorbidity. For the groups with psychiatric comorbidity, the most pronounced deterioration of QOL with increasing frequency of ES was observed in patients with depressive and dissociative disorders. A number of authors also emphasize that high seizure frequency may be associated with worse QOL [27] and that overall QOL scores are statistically significantly higher in the group of patients with a good response to ASM, compared to those who do not respond well to applied ASMs [28] and have poor ES control [27]. Other authors did not confirm the determining role of ES frequency for worse QOL. Luoni et al. summarizing the results of the study of the outcome of pharmacoresistance in epilepsy (SOPHIE) in a population of children and adults with pharmacoresistant epilepsy, emphasize that the results of the adverse event profile questionnaire (greater toxicity of ASMs) were the strongest predictors for all QOLIE-31 subscales except for the overall QOL subscale, where the Beck depression inventory was the strongest predictor. The patient’s assessment of both the overall tolerability and the overall efficacy of the treatment significantly influenced the overall QOL score [29]. AEs of ASMs are one of the strongest predictors of impaired QOL, independent of the impact of ES. AEs impair QOL through the impact of ASMs on behavior, cognition, and physical and mental functioning.

The relationship between psychiatric disorders and epilepsy is complex and raises the question of whether psychiatric disorders are comorbidities simply associated with treatment-resistant ES or are directly involved in the development of treatment resistance [30]. Whether treatment of comorbid psychiatric disorders can affect seizure frequency remains to be determined [13]. In many cases, psychiatric symptoms are considered consequences of epilepsy rather than comorbidities that deserve special attention and treatment. All these data emphasize the need for an initial assessment of psychiatric symptoms for optimal treatment in the presence of ES, especially in atypical or subsyndromal courses of psychiatric comorbidity [20].

Study limitations

One of the limitations of our study is the small sample sizes for the individual groups of patients with psychiatric comorbidity. Another possible limitation is related to the effects and interactions of antidepressants and ASMs in patients with psychiatric disorders, considering their dosage and duration of administration. We also did not analyze the temporal relationship between the onset of the epileptic disease and the psychiatric symptoms.

## Conclusions

The main conclusion of our study is the presence of an interaction between psychiatric comorbidity, the clinical course of the disease, and the deteriorated QOL in patients with epilepsy. Psychiatric comorbidity negatively influenced the presence of ES and AEs and was associated with lower scores for all aspects of QOL of patients with epilepsy, with those related to physical problems, emotional well-being, and cognitive functions being most significantly affected. Further attention, comprehensive care, and targeted research are needed to analyze individual psychiatric comorbidities in patients with epilepsy for early detection and treatment.
